# Mild cold induced thermogenesis: are BAT and skeletal muscle synergistic partners?

**DOI:** 10.1042/BSR20171087

**Published:** 2017-09-28

**Authors:** Naresh C. Bal, Santosh K. Maurya, Sunil Pani, Chinmayee Sethy, Ananya Banerjee, Sarita Das, Srinivas Patnaik, Chanakya N. Kundu

**Affiliations:** 1 School of Biotechnology, KIIT University, Campus-11, Bhubaneswar, Odisha 751024, India; 2 Department of Physiology and Cell Biology, The Ohio State University, College of Medicine, Columbus, OH 43210, U.S.A.; 3 Sanford Burnham Prebys Medical Discovery Institute, Lake Nona, Orlando, FL 32827, U.S.A.

**Keywords:** brown adipose tissue, Core body temperature, cold adaptation, cytokines, skeletal muscle, thermogenesis

## Abstract

There are two well-described thermogenic sites; brown adipose tissue (BAT) and skeletal muscle, which utilize distinct mechanisms of heat production. In BAT, mitochondrial metabolism is the molecular basis of heat generation, while it serves only a secondary role in supplying energy for thermogenesis in muscle. Here, we wanted to document changes in mitochondrial ultrastructure in these two tissue types based upon adaptation to mild (16°C) and severe (4°C) cold in mice. When reared at thermoneutrality (29°C), mitochondria in both tissues were loosely packed with irregular cristae. Interestingly, adaptation to even mild cold initiated ultrastructural remodeling of mitochondria including acquisition of more elaborate cristae structure in both thermogenic sites. The shape of mitochondria in the BAT remained mostly circular, whereas the intermyofibrilar mitochondria in the skeletal muscle became more elongated and tubular. The most dramatic remodeling of mitochondrial architecture was observed upon adaptation to severe cold. In addition, we report cold-induced alteration in levels of humoral factors: fibroblast growth factor 21 (FGF21), IL1α, peptide YY (PYY), tumor necrosis factor α (TNFα), and interleukin 6 (IL6) were all induced whereas both insulin and leptin were down-regulated. In summary, adaptation to cold leads to enhanced cristae formation in mitochondria in skeletal muscle as well as the BAT. Further, the present study indicates that circulating cytokines might play an important role in the synergistic recruitment of the thermogenic program including cross-talk between muscle and BAT.

## Introduction

Mitochondria are very dynamic organelles that undergo dramatic remodeling in response to increase in local energy demand within a cell. The mitochondrial architecture (including cristae density, compactness, length, shape, and size) is a reflection of their level of activity, and thus it is also an indicator of cellular energy status. It is believed that organs involved in thermogenesis within the mammalian body elevate their metabolism in response to cold adaptation. Two well-known sites of thermogenesis are brown adipose tissue (BAT) and skeletal muscle [1–5]. Both tissues use energy substrates such as fatty acids and glucose to convert the chemical energy generated in cells into heat as well as utilizing it for core body temperature (Tc) maintenance. BAT expresses a protein called uncoupling protein 1 (UCP1) that dissipates the mitochondrial proton gradient leading to heat generation [6–9]. On the other hand, skeletal muscle possesses multiple mechanisms of heat production including shivering and nonshivering thermogenesis (NST) [10–14]. These mechanisms employ futile ATP hydrolysis by various ATPases [15–20]. Hence, BAT-based thermogenesis is directly reliant on mitochondrial metabolism, whereas skeletal muscle based thermogenesis is indirectly linked to mitochondrial metabolism.

Utilization of these thermogenic processes enables mammals to maintain a constant Tc, usually approximately 37°C in spite of fluctuation in the temperature of the surrounding environment. From several earlier studies, it is clear that adaptation to different degrees of cold progressively recruits BAT, while other thermogenic sites are still poorly explored [1,18,21]. These studies showed that cold exposure results in increase in whole-body oxygen and food consumption. Morphological changes in the muscle tissue (increased red coloration) due to increased oxidative capacity and vascularization were also seen [18,22]. It has been reported that cold adaptation causes elevation in substrate uptake by both BAT and skeletal muscle [23–26]. Furthermore, some studies have shown that oxygen consumption (VO_2_) and mitochondrial enzymatic activity is progressively up-regulated in skeletal muscle upon adaptation to increasing cold [15,27–30]. However, changes in mitochondrial architecture at the ultrastructural level have not been adequately characterized with increasing degrees of cold adaptation.

Here, we provide ultrastructural evidence to the cold-induced tissue remodeling of BAT and skeletal muscle. Additionally, we were interested in characterizing changes in mitochondrial architecture in both tissues after adaptation to increasing degrees of cold. For the present study, normal adult C57BL/6J mice generated at thermoneutrality (29°C) were acclimatized to mild cold (16°C) and severe cold (4°C) for 15 days each. Acclimatization to 16°C was chosen as it has been shown that this shift from thermoneutrality activates NST, without evoking shivering [31]. We employed indirect calorimetry to measure whole-body metabolic changes and used extensive electron microscopic analysis to characterize ultrastructural changes. Data presented here shows that thermogenic load initiates elaborate cristae formation in the mitochondria correlating with the degree of cold exposure in both BAT and skeletal muscle. The co-ordination between these two major sites of thermogenesis during cold adaptation is important to maximize chances of survival while utilizing the least amount of energy expenditure. Several recent reports have suggested possible role of circulating cytokines in the relative recruitment of BAT compared with skeletal muscle during adaptive thermogenesis [32–36]. Hence, we have explored the effect of cold challenge on various circulating cytokines. Data presented here suggest that fibroblast growth factor 21 (FGF21), tumor necrosis factor α (TNFα), and interleukin 6 (IL6) may play a critical role in co-ordinating cold-mediated physiological adaptations, which has implication for cross-communication between muscle and BAT.

## Materials and methods

### Animals

Wild-type C57BL/6J male mice aged 12 weeks were utilized for the present study. The Ohio State University Institutional Animal Care and Use Committee approved the study protocol. All the animal procedures were carried out at an AAALAC-accredited animal facility and conducted in accordance with the Guide for the Care and Use of Laboratory Animals. Mice were housed in groups of five in polycarbonate cages containing bedding (Andersons/Bed-o’cobs, Maumee, OH) and maintained in a controlled environment at a temperature of 29 ± 1.0°C with a 12:12-h light:dark cycle and arelative humidity of approximately 50%. Mice were fed with regular rodent diet (2014, Harlan Teklad, Madison, WI) and provided with water *ad libitum*.

### Metabolic measurements and acute cold challenge

The VO_2_ and physical activity were measured continuously using Comprehensive Lab Animal Monitoring System (oxymax/CLAMS) equipped with temperature-controlled environmental chamber from Columbus Instruments, Columbus, OH throughout the cold adaptation. First, we measured the VO_2_ in mice at 29 ± 1°C to determine the basal metabolic rate (BMR) for 5 days. Then, the ambient temperature was dropped to 16 ± 1°C and maintained for 15 days before further decreasing the temperature to 4 ± 1°C and maintained for additional 15 days. The Tc was monitored three times daily using thermal transponders, IPTT300 (Bio Medic Data Systems, Seaford, DE), implanted in backs of the mouse below the skin. Food intake and body weight were measured every alternate day.

### Tissue histology and EM

BAT was fixed in 10% formalin and embedded in paraffin and were sectioned at 5 μM on a microtome. The slides were air dried overnight. Frozen skeletal muscle samples were sectioned at 10 μM on a Leica 1900 cryostat and were allowed to come to room temperature (RT). Both BAT and skeletal muscle slides were stained with Hematoxylin and Eosin (H&E) using the Thermo Shandon Sequenza System at RT. The slides were scanned using the Leica Aperio Scanscope XT using the 20x/0.75 Plan Apo Olympus objective and compound microscope. For TEM, samples of BAT and longitudinal sections of skeletal muscles were taken after the indicated period of cold adaptation and fixed with 1% glutaraldehyde solution. The samples were processed by the core facility. Electron micrographs were obtained using a Tecnai G2 Spirit transmission electron microscope (FEI, Hillsboro, OR). For measuring mitochondrial abundance, muscle samples (at least 50 micrographs) collected from three different animals were analyzed.

### Immunostaining for Nectin-4

IHC of Nectin-4 (Abcam, U.S.A., cat# ab57873) was carried out using kit (Vector Lab, cat# SK-4100) as per the protocol mentioned earlier [37]. Briefly, the slides were deparaffinized by heating at 60°C followed by immersing in xylene for 5 min. The samples were rehydrated with decreasing percentages of alcohol (100–50%) for 5 min each. Next, antigen retrieval was carried out by heating the slides in citrate buffer solution for 20 min. The slides were allowed to cool down, washed with 1× PBS and were blocked with 5% FBS for 20 min. Slides were then subjected to peroxide blocking for another 10 min and followed by incubation with anti-Nectin-4 antibody for 4°C overnight. Slides were then washed with 1× PBS followed by incubation with secondary antibody conjugated with horseradish peroxidase (HRP) for 2 h and developed with 3,3′-Diaminobenzidine (DAB). Slides were counterstained with Hematoxylin and images were captured using Leica bright field microscope.

### Cytokine/hormone measurement

Blood was collected by cardiac puncture from the animals after killing; plasma was separated and stored at −80°C until use. The levels of cytokines and chemokines were measured using magnetic bead based immunology multiplex assay kits procured from Merck Millipore, U.S.A. following manufacturer’s protocol. IL1α, IL6, and TNFα were measured using ‘MCYTOMAG-70K’ and peptide YY (PYY), leptin, insulin, and amylin were measured using ‘MMHMAG-44K’. Data were recorded and analyzed on a Luminex xMAP system using the software program ‘xPONENT’. Levels of FGF21 were measured using 96-well ELISA-based assay kit (EZRMFGF21-26K) from EMD Millipore Corporation, MA, U.S.A. following the protocol provided along with the kit. Six samples per group were analyzed. Plasma levels of VEGF were measured using quantitative ELISA kit by R&D Systems, ME, U.S.A. following the protocol provided by the manufacturer.

### Statistical analysis

Physical activity was analyzed taking only the counts of breakage of IR beam to detect movement of the mice in *xy*-axis. Metabolic data from six mice was analyzed together per group. Two-way ANOVA and/or Student’s *t* test was used for the determination of statistically significant difference.

## Results and discussion

Research on defining mechanisms of NST has gained significant attention in the last decades with hopes of unraveling pharmacological targets to counter metabolic disorders [4,38–41]. This has resulted in identification of several novel mechanisms of NST [16,17,19,42–43]. Interestingly, all the NST mechanisms are intricately associated with mitochondrial metabolism, ultimately connected to mitochondrial architecture. In the present study, we set out to define the ultrastructural remodeling of mitochondria in the skeletal muscle and BAT upon cold adaptation.

### Cold adaptation increases whole-body metabolic rate

**Figure 1:**
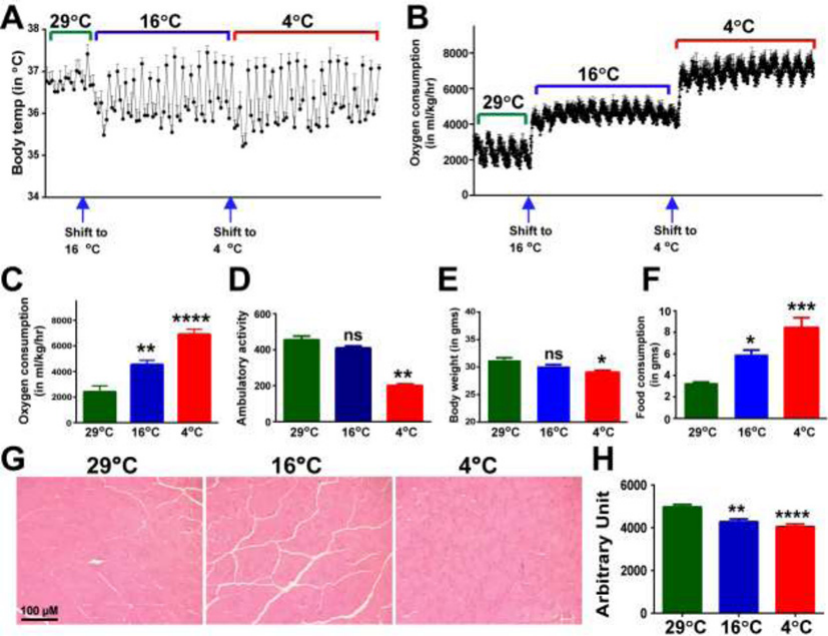
Effect of mild and severe cold adaptation on physiological parameters and skeletal muscle structure (**A**) Tc of mice was ∼1°C lower than the average Tc at thermoneutrality in the first 6–10 h. After an initial drop in Tc, the mice were able to up-regulate and maintain Tc quite close to optimal. (**B**,**C**) VO_2_ of mice during cold challenge shifts from that maintained at ambient temperature as indicated by arrow below the graph. (**D**) Severe, but not mild cold exposure impairs degree of ambulatory movement of the mice. (**E**) Adaptation to severe cold caused a greater decrease in body weight. (**F**) A significant increase in food intake was observed in both mild and severe cold conditions (**G**,**H**) Representative cross-sections of skeletal muscle fiber stained with H&E. Fiber cross-sectional area decreases after cold adaptation.

As expected, all the mice were able to maintain Tc close to optimal (37°C) throughout the period of cold challenge. However, just after shifting from ambient temperature, we observed a drop in Tc of approximately 1.0°C for a few hours (Figure 1A). A rapid increase in VO_2_ was recorded indicating a swift rise in the metabolic rate upon cold challenge (Figure 1B,C). Enhanced O_2_ consumption upon cold adaptation was also observed in several other organisms, including birds, mammals, and fishes which may function to maintain the supply of oxygen and energy to aerobically active tissues [18,28,44,45]. At thermoneutrality, the VO_2_ was 2411 ± 462.3 ml/kg/h, while an increase by 88% to 4538 ± 333.1 was recorded during the adaptation to 16°C. Interestingly, adaptation to mild cold did not elicit any significant change in long-range physical activity (454.8 ± 21.69 compared with 409.4 ± 11.52) and body weight (31.32 ±0.93 compared with 30.2 ± 0.6), while food consumption (3.33 ± 0.077 g/mice/day compared with 5.98 ± 0.36 g/mice/day) was elevated significantly (Figure 1D–F). A reduction in body mass was reported after 4 weeks of cold acclimation in ducklings [13]. The increased VO_2_ and food intake suggested up-regulation of thermogenesis. The data showing similar level of long-range physical activity compared with thermoneutrality suggest that NST is a major component of energy expenditure during mild cold adaptation. In contrast, severe cold adaptation resulted in a modest decrease in body weight to 29.05 ± 0.82 and approximately three-fold up-regulation in VO_2_ (6904 ± 397.6) and food consumption (8.45 ± 2.24) indicating a significant high energy cost of thermogenesis. In addition, it was found that long-range physical activity was much lower than under mild cold to 200.6 ± 8.02. During shivering, mice cannot perform long-range physical activity; hence these data may suggest that shivering is important during severe cold, while its role during mild cold might be minimal. Taken together these organismal data indicate that NST is the primary mechanism of muscle thermogenesis, especially during mild cold, and corroborates recent study on humans that suggested muscle thermogenesis is not limited to shivering [46]. We chose to perform H&E staining on predominantly fast-twitch skeletal muscles (quadriceps and tibialis anterior (TA)). The data show that adaptation to even mild cold leads to significant reduction in fiber cross-sectional area of muscles (Figure 1G,H). The average size of fiber at 29, 16, and 4°C were 4967 ± 117.1, 4277 ± 127.5, and 4040 ± 120.1, respectively; which is reduction in 13.38% after mild and 18.66% after severe cold adaptation. These data are in agreement with data published previously for other rodent species [47].

### BAT is significantly modified during cold adaptation even in gross structure

**Figure 2:**
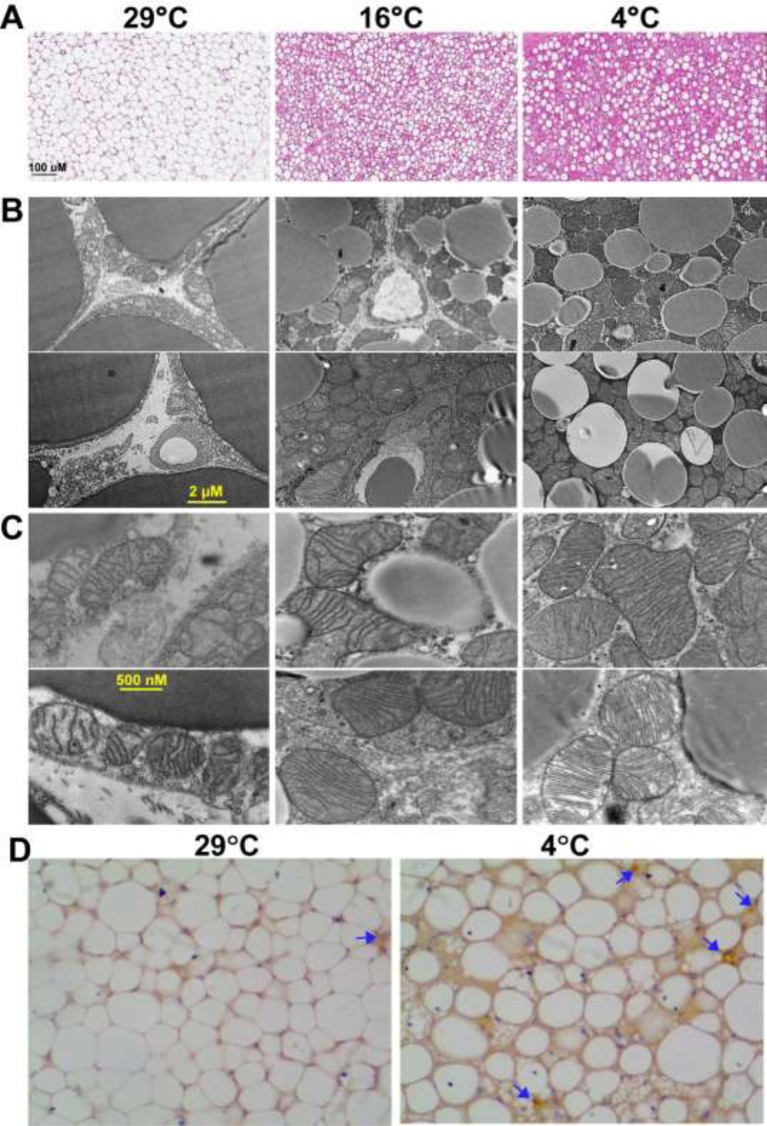
Changes in BAT upon adaptation to cold (**A**) Representative images of histological sections of BAT stained with H&E. Cold adaptation caused depletion of lipid droplets in BAT adipocytes. Two representative images of TEM of BAT sections were taken at both lower (**B**) and higher magnifications (**C**); housing temperature is mentioned on the top of the H&E images. (**D**) Staining with anti-nectin4 antibody shows increased expression after adaptation to severe cold (4°C).

As BAT is the major site of NST; we carefully analyzed the structural and morphological changes in this tissue during cold adaptation. When mice are reared at thermoneutrality, the BAT is quite less prominent, pale brown in color and surrounded by a large volume of white adipose tissue (WAT). Upon H&E staining, most of the adipocytes in the BAT are filled with lipid droplets and appear mostly unilocular (Figure 2A). Imaging with TEM showed that the BAT cells possess only a few mitochondria located close to the plasma membrane (Figure 2B,C). Further, these mitochondria contained only few and irregularly shaped cristae (Figure 2C). After adaptation to mild cold, several changes in the BAT were observed. WAT surrounding BAT reduced in quantity and BAT became browner in color making it easily distinguishable from WAT. Staining with H&E showed that in most BAT adipocytes the intracellular lipid depots were depleted (Figure 2A), which correlates with increase in UCP1 expression in the BAT reported after adaptation to mild cold [1]. TEM analysis showed that BAT adipocytes are filled with more mitochondria that are located all over the cells (Figure 2C). In higher resolution, the mitochondria showed more regular cristae structure. In contrast, adaptation to severe cold brought about more prominent changes. Visually, almost no WAT was found around the BAT and H&E staining showed small lipid droplets inside the adipocytes (Figure 2A). Browning of white adipocytes (especially in inguinal WAT) caused from mitochondrial remodeling has been reported to play a part in adaptive thermogenesis [43,48,49]. TEM analysis showed that mitochondria became heavily loaded with the cristae. Taken together, these data suggest that cristae density is low in mitochondria in BAT under thermoneutrality but increases during cold adaptation. Cold-induced NST in BAT is reliant upon significant enhancement of vascularization, which is reported to be mediated by VEGF-signaling [50,51]. Here we studied the involvement of a novel VEGF-independent mediator of neovascularization called ‘Nectin 4’ in BAT. We found that Nectin4 expression is up-regulated during cold adaptation. This indicates the role of Nectin4 in increasing vascularization of BAT (Figure 2D).

### Cold adaptation enhances mitochondrial abundance and cristae density in the fast-twitch skeletal muscle

Next, we decided to study the structural and ultrastructural changes in fast-twitch skeletal muscle as we noted a reduction in fiber cross-sectional area. Earlier cold adaptation studies have suggested that mitochondrial number in the skeletal muscle increases during adaptation to severe cold [15,52,53]. Here, we analyzed mitochondrial abundance in quadriceps muscle after mild cold adaptation by using TEM.

**Figure 3:**
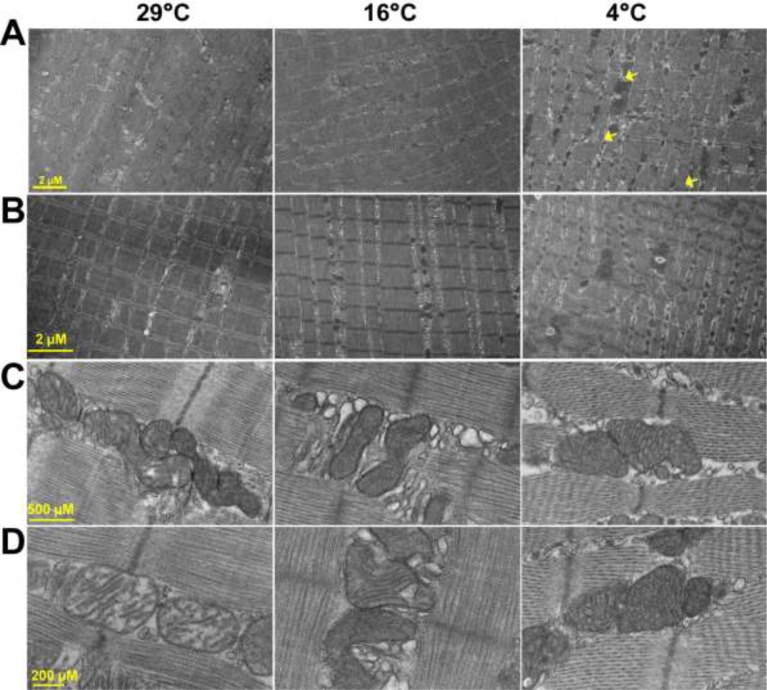
Ultrastructural changes in the skeletal muscle due to cold adaptation (**A**–**D**) When mice are adapted to thermoneutrality, the number of intermyofibrilar mitochondria is low with fewer cristae, indicating ultrastructurally ill developed mitochondria. However, adaptation to cold increases abundance of mitochondria in the intermyofibrilar region of the quadriceps muscle. Higher magnification images show increased cristae density after adaptation to both mild and severe cold adaptation. Occasionally, lipid droplets were found in the intermyofibrilar area pointed by yellow arrows.

We selected quadriceps as we have earlier observed alteration in several (calcium handling and mitochondrial) proteins [15,18]. Here, we observed that when mice are adapted to thermoneutrality, their muscles contain lower numbers of intermyofibrilar mitochondria. The ultrastructure of these mitochondria showed very few cristae (Figure 3). As adaptation to mild cold brings about increase in the abundance in intermyofibrilar mitochondria, we analyzed carefully the architecture of this mitochondrial subpopulation. Interestingly, lipid droplets were also observed more often in the intermyofibrilar region of fast-twitch skeletal muscles such as quadriceps and TA, which supports earlier findings that cold acclimatization promotes fatty acid metabolism in the skeletal muscles (Figure 3A,B). Images at higher magnifications suggest that cristae density increases with adaptation to progressive degrees of cold exposure (Figure 3C,D). Although intermyofibrilar mitochondrial number did not increase in predominantly slow-twitch skeletal muscles such as soleus and diaphragm, the cristae density was found higher upon adaptation to severe cold (data not shown). Several groups have previously reported that cristae density, mitochondrial oxidative metabolism, and biogenesis all increase in the skeletal muscles of various species after cold adaptation [44,54,55]. These data suggest that mitochondrial architecture in the muscle is intricately associated with the thermogenic demand on the body.

### Cytokines as possible mediators of cold-induced adaptations

**Figure 4:**
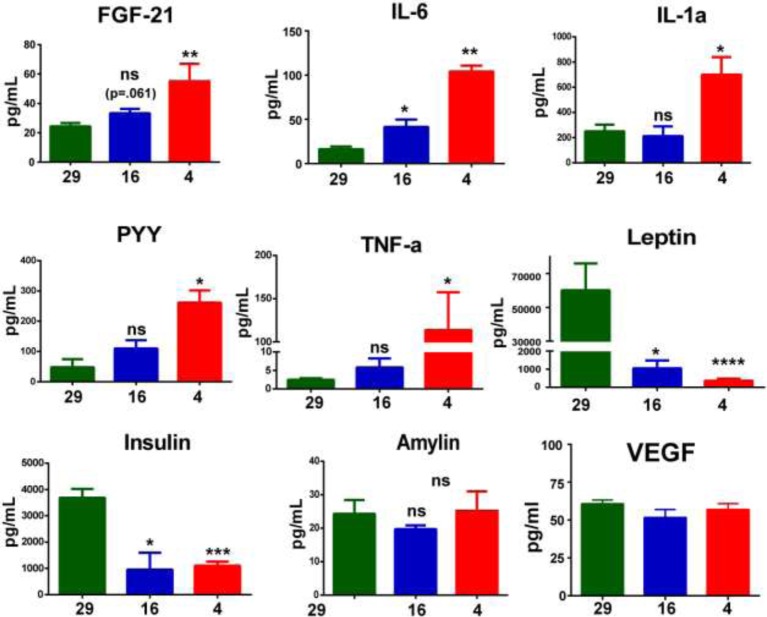
Plasma level of cytokines in mice adapted to different ambient temperatures FGF21, IL6, IL1α, PYY, TNFα, leptin, insulin, amylin, and VEGF were measured. Data were analyzed using two-way multiple ANOVA. Comparison between FGF21 levels at thermoneutrality and mild cold was performed by Student’s *t*test showed a trend for up-regulation although not statistically significant.

An increasing number of publications suggest a possible cross-talk between skeletal muscle and BAT via humoral-mediated factors including cytokines in regulation of energy homeostasis. So, the role of cytokines in orchestrating the adaptive changes in the mammalian body during cold acclimatization might be important, but has not been studied. Therefore, we measured the serum levels of several cytokines and hormones in the mice acclimatized to different temperatures. Interestingly, serum level of FGF21 was up-regulated by 47.3% after mild cold and by approximately 2.2-fold after severe cold. Mild cold adaptation resulted in a significant up-regulation (1.5-fold) of IL6 as was observed in other organisms [56,57]. In addition, the serum of mice acclimatized to severe cold exhibited higher levels of IL6 (approximately 6.4-fold), IL1α (approximately 2.8-fold), PYY (approximately 5.2-fold), and TNFα (approximately 100-fold) in comparison with thermoneutrality (Figure 4). In contrast, leptin showed most remarkable down-regulation: 61.2-fold and 168.5-fold after mild and severe cold adaptation, respectively. Down-regulation of insulin was also observed upon adaptation to mild and severe cold. However, we did not find any change in the level of amylin due to cold adaptation. Interestingly serum level of VEGF, a key mediator of neovascularization, does not differ significantly with even severe cold exposure. Any change in the expression of VEGF receptors at the thermogenic sites can up-regulate VEGF-dependent vascularization, which is yet to be fully described. Leptin and PYY are important regulators of feeding behavior and might be involved in cold-induced hyperphagia [58]. Role of IL6 and insulin might be to co-ordinate substrate utilization in thermogenic tissues and lipolysis from WAT [59]. Previously, it has been reported that TNFα production from CD4^+^ cells is down-regulated after cold adaptation [58]. Our data showing up-regulation plasma TNFα level suggest that upon cold, TNFα is secreted from a source other than immune cells. The role of FGF21 in cross-communication between BAT and muscle has been proposed [35,36]. A recent study showed that cold-adapted UCP1-knockout mice secrete higher amounts of FGF21 [32]. Therefore, up-regulation of FGF21 levels might play a critical role in relative recruitment of BAT and skeletal muscle.

### Evolutionary implication

BAT is an energetically efficient thermogenic system. But, it is small and localized, meaning the heat needs to be circulated throughout the body for the maintenance of the Tc, which in turn can be costly. In contrast, skeletal muscle based NST is less efficient than BAT. However, muscles being distributed in almost all parts of the body can provide a mechanism of heat production that is scattered throughout the body. As BAT provides a centralized heating system and skeletal muscle allows heat production *in situ* in various parts of the body, a unique combination of these two systems of heat production can reduce the energy cost of whole-body temperature maintenance. Minimization of energy cost of endothermy reduces the demands on diet intake, provides the opportunity to invade new geographic areas with lesser food availability and longer spells of cold weather [3]. Additionally, swift co-ordination between these two isolated compartments of thermogenesis may have a selective advantage for turning either of them on or off, depending on thermogenic demand. We propose that cytokines are utilized to mediate synergistic recruitment of BAT and muscle and their signaling might have an evolutionary importance in the development of endothermy amongst eutherian mammals.

## Conclusion

Data presented here suggest that mitochondrial architecture at both thermogenic sites is closely associated with the degree of cold exposure. In addition to shivering, skeletal muscle metabolism also plays a key role in NST. The mechanism of activation of NST in the skeletal muscle needs more precise understanding. Our data suggest that cytokines, especially FGF21, might be a potential determinant of relative recruitment of BAT and skeletal muscle. A better understanding of how NST mechanisms in skeletal muscle operate may provide pharmacological targets to increase whole body energy expenditure to counter metabolic diseases such as obesity and type II diabetes. Toward this understanding, a few key questions that should be addressed by future studies, including: identifying molecular regulators of mitochondrial cristae formation in BAT and skeletal muscles; delineating the signaling mechanism that links the energy demand of the body with mitochondrial cristae enrichment; identifying the role of adipokines/myokines in energy sensing and their subsequent recruitment during high-energy demanding conditions like cold and exercise; describing the muscle activity dependent secretion of myokines in co-ordination of energy homeostasis; and understanding the role of cytokines in mitochondrial architecture in thermogenic organs.

## Abbreviations

BAT: brown adipose tissue
FGF21: fibroblast growth factor 21
H&E: hematoxylin and eosin
IL6: interleukin 6
NST: nonshivering thermogenesis
PYY: peptide YY
RT: room temperature
TA: tibialis anterior
Tc: core body temperature
TNFα: tumor necrosis factor α
UCP1: uncoupling protein 1
VEGF: vascular endothelial growth factor
VO_2_: oxygen consumption
WAT: white adipose tissue
