# Expression of Concern: Mild cold induced thermogenesis: Are BAT and skeletal muscle synergistic partners?

**DOI:** 10.1042/BSR-2017-1087T_EOC

**Published:** 2021-04-07

**Keywords:** brown adipose tissue, cold adaptation, Core body temperature, cytokines, skeletal muscle, thermogenesis

The Editorial Office has been made aware of potential issues surrounding the scientific validity of this paper, hence has issued an expression of concern to notify readers whilst the Editorial Office investigates.

